# Impact of an educational intervention on standard precautions during the COVID-19 pandemic

**DOI:** 10.1590/0034-7167-2022-0750

**Published:** 2023-09-04

**Authors:** Natália Liberato Norberto Angeloni, Maria Heloísa do Nascimento Silva, Lomberto Ariel Romeu Valle, Álvaro Francisco Lopes Sousa, Marília Duarte Valim, Denise de Andrade, Inês Fronteira, Aires Garcia dos Santos

**Affiliations:** I Universidade Federal do Mato Grosso do Sul. Três Lagoas, Mato Grosso do Sul, Brazil; II Hospital Sírio Libânes, Instituto de Ensino e Pesquisa. São Paulo, São Paulo, Brazil; III Universidade Nova de Lisboa, Instituto de Higiene e Medicina Tropical. Lisboa, Portugal; IV Universidade Federal do Mato Grosso. Cuiabá, Mato Grosso, Brazil; V Universidade de São Paulo. Ribeirão Preto, São Paulo, Brazil

**Keywords:** Universal Precautions, Nursing Professionals, Knowledge, Nursing, Continuing Education in Nursing, Precauciones Universales, Profesionales de Enfermería, Conocimiento, Enfermería, Educación Continuada en Enfermería, Precauções Universais, Profissionais de Enfermagem, Conhecimento, Enfermagem, Educação Continuada em Enfermagem

## Abstract

**Objective:**

To evaluate the impact of an educational intervention on the knowledge of nursing professionals regarding standard precautions.

**Methods:**

This is an almost experimental study conducted with 100 nursing professionals. Data collection was performed using a sociodemographic characterization instrument and the Standard Precautions Knowledge Questionnaire. The educational intervention was based on five moments, where the approach to questions with less than 70% accuracy was intensified.

**Results:**

There was a significant difference between the scores of healthcare professionals before (16.20 ± 1.51) and after (16.90 ± 1.31) the educational intervention (W=3.336; p < 0.05). Regarding knowledge about hand hygiene after glove use, an increase in knowledge from 83% to 93% was obtained.

**Conclusions:**

A positive effect on the professionals’ knowledge was recorded, demonstrating advances regarding the strengthening of already acquired knowledge and the understanding of new knowledge.

## RESUMO

**Objetivo:**

avaliar o impacto de uma intervenção educativa no conhecimento dos profissionais de enfermagem em relação às precauções padrão.

**Métodos:**

trata-se de um estudo quase-experimental, realizada com 100 profissionais de enfermagem. Para a coleta de dados, foram utilizados o instrumento de caracterização sociodemográfica e o questionário de Conhecimento das Precauções-Padrão. A intervenção educativa foi baseada em 05 momentos, onde se intensificou a abordagem em questões com menos de 70% de acerto.

**Resultados:**

Houve diferença significativa entre os escores dos profissionais de saúde antes (16,20 ± 1,51) e após (16,90 ± 1,31) a intervenção educativa (W=3,336; p < 0,05). Em relação ao conhecimento sobre a higiene das mãos após uso de luvas, obteve-se um aumento no conhecimento de 83% para 93%.

**Conclusões:**

registrou-se efeito positivo no conhecimento dos profissionais, demonstrando avanços no que diz respeito ao fortalecimento dos conhecimentos já adquiridos e à compreensão de novos saberes.

## RESUMEN

**Objetivo:**

Evaluar el impacto de una intervención educativa en el conocimiento de los profesionales de enfermería sobre las precauciones estándar.

**Métodos:**

Este es un estudio cuasiexperimental realizado con 100 profesionales de enfermería. Para la recopilación de datos, se utilizaron un instrumento de caracterización sociodemográfica y el cuestionario de Conocimiento de las Precauciones Estándar. La intervención educativa se basó en cinco momentos, en los cuales se intensificó el abordaje de cuestiones con menos del 70% de aciertos.

**Resultados:**

Hubo una diferencia significativa entre las puntuaciones de los profesionales de salud antes (16,20 ± 1,51) y después (16,90 ± 1,31) de la intervención educativa (W = 3,336; p <0,05). En relación con el conocimiento sobre la higiene de las manos después del uso de guantes, se obtuvo un aumento en el conocimiento del 83% al 93%.

**Conclusiones:**

Se registró un efecto positivo en el conocimiento de los profesionales, demostrando avances en cuanto al fortalecimiento de los conocimientos ya adquiridos y la comprensión de nuevos saberes.

## INTRODUCTION

Healthcare-associated infections (HAIs) have various impacts on patient morbidity and mortality as well as healthcare costs, making it a serious global public health problem. An effective strategy for reducing HAIs and improving the quality of care is the proper use of standard precautions (SPs) ^(1)^.

SPs provide safety and protection against biological risks. They involve the correct use of gloves, gowns, eye protection, hand hygiene, proper disposal of sharp objects, and cough etiquette^(2-3)^. In addition, the Center for Disease Control and Prevention (CDC) has added other prevention measures such as respiratory hygiene or cough etiquette, safe injection practices, and the use of protective masks for inserting catheters or injections involving lumbar punctures^(4)^. However, there is a lack of knowledge among professionals regarding the correct use of SPs^(5)^.

Several factors are directly related to the deficit in knowledge and adherence to SPs among professionals, such as management support for safer work practices, low perception of prevention efficacy due to inadequate knowledge of the importance of SP use, unavailability and inaccessibility of personal protective equipment (PPE), lack of safety performance feedback, among others. Another important aspect to highlight is that the low knowledge of SPs may also be related to the fact that most nurses do not receive training at the beginning of their work activities in healthcare institutions^(6)^.

Thus, training has a significant and effective influence on the attitude and knowledge of professionals in performing their work activities safely, increasing awareness of safety protocols^(1,7)^. With the onset of the COVID-19 pandemic and the growing global spread of the virus, significant changes have been observed in the production and distribution capacity of PPEs, resulting in incompatibility in the availability of some materials and compromising compliance with use^(8)^. This atypical scenario required regulatory health agencies to issue new recommendations, such as surgical masks should be snugly secured on the face, eye protection should be used for all patient care encounters, and suspected or confirmed COVID-19 cases should use a respirator such as an N95 or PFF2 with eye protection, even when there is no exposure to aerosols, as recommended by the CDC ^(9-10)^.

Although the use of PPE is not a recent recommendation, the transmission and spread mechanisms of COVID-19 required the development of new technical-scientific knowledge aimed at promoting safety in healthcare services ^(3-6)^. In this context, the high demand for care for COVID-19 patients, the overload of care, and the deficit in knowledge are factors that contributed to the high turnover of nursing professionals during the COVID-19 epicenter ^(7-10)^.

However, the proper use of PPEs does not eliminate the risks of exposure, but they are reduced with correct use, thereby reducing the propensity for work-related accidents. Failure to use PPEs exposes both the professional and the patient to infectious diseases^(9)^.

Offering training to professionals seeks to expand knowledge and promote correct practices regarding the use of SPs, thus minimizing the dissemination of pathogens^(6)^. The importance of permanent education that promotes biosafety from the beginning of professional education is highlighted as a strategy to reduce the impacts of lack of knowledge when professionals are exercising their functions since work-related accidents may be related to a lack of knowledge^(10)^.

## OBJECTIVE

To evaluate the impact of an educational intervention on the knowledge of nursing professionals regarding standard precautions.

## METHODS

### Ethical aspects

The study was conducted in accordance with national and international ethical guidelines and approved by the Research Ethics Committee of the Federal University of Mato Grosso do Sul (UFMS), whose opinion is attached to this submission. Informed consent was obtained in writing from all individuals involved in the study.

### Type of study

Quasi-experimental research, with a before-and-after design of a single group guided by the CONSORT tool. The essential characteristic of this investigation is that the researchers control and manipulate the conditions they are interested in, meaning they cause a change in the value of an independent variable and observe the effect of that change on another dependent variable^(11)^.

### Study location

The study was conducted in the city of Três Lagoas, in the east of Mato Grosso do Sul, Brazil, in a hospital that currently serves as a reference for 10 municipalities in the state. The hospital has 166 existing beds, 90% of which are dedicated to public and free care through the Brazilian Unified Health System (SUS). The estimated population of the municipality is 123,281 inhabitants.

### Inclusion and exclusion criteria

The nursing team at the institution had a total of 358 active nursing professionals, of whom 149 were eligible to participate. Professionals who were in positions that did not have direct contact with the patient during data collection were excluded.

### Instruments

Initially, a sociodemographic characterization instrument was applied for variables such as gender, professional category, sector, age range, work time range at the hospital, and hours worked per week^(12)^. To measure knowledge, the Standard Precautions Knowledge Questionnaire (QCPP) was used, created by Chinese researchers^(13)^ and adapted to the Portuguese language in Brazil ^(14-16)^, with satisfactory validity indexes. Stability was calculated using the intraclass correlation coefficient (ICC), with a value of 0.91, and agreement was tested using the Kappa coefficient, with a “perfect” substantial classification for all items on the instrument.

The self-administered questionnaire has 20 questions, where each correct answer is scored 1 point, and each “I don’t know” or incorrect answer is scored 0 points. The possible score ranges from 0 to 20 points, and the higher the score, the greater the professional’s knowledge related to Standard Precautions. The use of the knowledge questionnaire can help in planning intervention actions aimed at patient and professional safety, with the aim of improving the use of Standard Precautions^(16)^.

### Methodological approach

The methodological approach used in this study was based on a previous study^(17)^ that conducted an educational intervention with healthcare professionals on standard precautions (SPs), consisting of 5 moments.

### Step 1 - Presentation of the Informed Consent Form (ICF) and Instruments

An initial approach was made with the nursing professionals who worked directly in the care, clarifying the research and obtaining authorization for participation through the Informed Consent Form (ICF). After acceptance, the instruments were answered by professionals during the shift through the Google Forms questionnaire link, using technological devices such as cell phones and tablets, with an average of 20 minutes for completion of all questionnaires.

Data collection for the first step occurred from February to March 2022; the educational intervention in April; and the second questionnaire application in May and June 2022. It is pertinent to highlight that, during this period, the pandemic status conferred by the World Health Organization (WHO) was still in effect, but without social isolation, mandatory use of masks, with a reduction in the number of deaths and severe cases, and an increase in vaccination rates.

### Step 2 - First phase of data collection

After acceptance of the professionals, the instruments were used (sociodemographic characterization instrument and knowledge evaluation questionnaires), and both instruments are self-reported by nursing professionals. Then, the answers to the instruments were analyzed in order to guide the construction of the educational intervention, with a focus on knowledge/practice.

The ICF and electronic forms were sent through the Google/Gmail platform, called Google Forms, and the study subjects were provided with the survey link, which contained the ICF page.

### Step 3 - Educational intervention

The presentation was built following the guidelines and recommendations proposed by the CDC^(18)^ for SPs for the care of all patients, and an educational video validated by expert professionals was used to report the importance of using SPs^(2)^.

In the third moment, the educational intervention was carried out, with an average duration of 30 minutes, in 4 periods, covering all shifts of the hospital professionals. It was based on the analysis of the results obtained in the knowledge questionnaire on SPs, through studies where the researcher asked the questions, exposing through the use of a Data Show, and the professionals answered afterwards.

For each question with a correct answer of less than 70%, the true answer based on the theoretical reference of the last 5 years of Brazilian and international literature was presented. A culture of educational and constant learning promotes the development of resilience, facilitating learning and improving professional readiness^(19)^.

It took place in April 2022, lasting three days, covering all shifts of the institution. The construction of the material was consulted through virtual databases, using the PowerPoint tool for better organization of information and exposure to collaborators, bringing, first, concepts related to HAI related to patient safety and types of SPs.

Further in-depth analysis was carried out on questions with a cutoff value of <70% in the first phase of data collection, for the construction of the intervention proposal, as recommended by the literature^(20-24)^. In order to strengthen knowledge and the importance of using SPs among nursing professionals, an educational video was used to compose the intervention material, a video built with the strategic objective of stimulating the adherence to SPs by nursing workers.

This same video was constructed through a descriptive study conducted with 197 nursing workers in Cuiabá, Mato Grosso, in 02 hospitals. For the construction of the video, data were collected through the Questionnaire for Knowledge and Compliance with Standard Precaution Scale (Portuguese version), to identify knowledge and factors that interfere with adherence to SPs. Afterwards, a methodological study was carried out and the video was structured based on the results obtained in the first phase of the research, following Nola Pender’s revised health promotion model. The video was validated using the Delphi technique and reviewed by 13 specialists. Therefore, the video was considered valid by the experts and could be used to expand and strengthen knowledge among nursing professionals, encouraging adherence to standard precautions^(2)^. Before conducting the educational intervention with the professionals, a presentation and discussion meeting was held with the nursing and medical coordination of the Hospital Infection Control Commission (CCIH) and the continuing education department of the institution to obtain their agreement, considering the hospital’s existing norms and routines on the topic.

### Step 4 - Reapplication of the Standard Precaution Knowledge Questionnaire

After the completion of the educational intervention, the standard precaution knowledge questionnaires were re-administered to analyze the impact on the knowledge of PPE among nursing professionals after the educational intervention. At this stage, the COVID isolation unit was already inactive due to the decrease in severe cases, which may be related to the vaccination coverage and isolation measures that were followed.

### Step 5 - Comparison of Obtained Data

Finally, a comparative analysis of the 1st and 4th moments was conducted in relation to knowledge before and after the educational intervention.

### Statistical Analysis

The scores obtained before and after the intervention were represented by mean and standard deviation. The Wilcoxon test was performed to evaluate possible significant differences. In addition, the score of each questionnaire was evaluated according to the sociodemographic characteristics of the sample. The Mann-Whitney and Kruskal-Wallis tests were performed to evaluate possible differences. Spearman’s correlation analysis was performed with the scores before and after the questionnaires with sociodemographic characteristics. The number of correct answers per question was evaluated according to the nominal variable “correct” and “incorrect”. To evaluate possible differences in the proportion of each questionnaire item, the Q-Cohrane test was performed. The Statistical Package for the Social Science (SPSS) software version 20.0 was used. The level of significance was set at 0.05.

Categorical variables were represented according to absolute and relative frequencies. To evaluate the difference between the responses before and after the intervention, the McNemar-Bowker test was performed. The statistical power of the sample was calculated using G Power software, considering a total of 99 individuals, an error rate (alpha) of 0.05, a bicaudal distribution area, and an effect size of 0.40. After the calculation, a 97.2% analysis power was obtained.

## RESULTS

Of the 100 nursing professionals who made up the final sample of the study, the majority were female (85.9%), and nursing technicians were the majority category of participants. The sociodemographic characterization data can be observed in Table 1.

**Table 1:** Sociodemographic characterization data of nursing professionals, Três Lagoas, Mato Grosso do Sul, Brazil, 2022

	n	%
Gender		
Female	85	85.9
Male	14	14.1
Professional category		
Nursing technician	77	77.8
Nurse	19	19.2
Nursing assistant	3	3.0
In which department do you work?		
Hospital Units	46	46.5
Intensive care unit (ICU)	21	21.2
Hemodialysis	15	15.2
Emergency room	14	14.1
Others	3	3.0
Age range		
From 20 to 40 years old	69	71.9
40 years or older	27	28.1
Length of time at the hospital		
From 0 to 10 years	87	88.8
From 11 to 20 years	7	7.1
From 21 to 30 years	4	4.1
> 30 years	0	0.0
Hours worked per week		
Up to 44 hours	70	71.4
>44 hours	18	18.4
Up to 30 hours	10	10.2

The Table 2 presented below shows the percentile of correct and incorrect answers for each question regarding the QCPP of professionals before and after educational intervention. It is possible to observe that most of the 20 questions had an increase in the percentage of correct answers, except for questions 10 and 12. However, statistically significant difference (p = 0.033) was demonstrated only in question 06.

**Table 2:** Relationship of correct and incorrect answers for each item of the Standard Precautions Knowledge Questionnaire, Três Lagoas, Mato Grosso do Sul, Brazil, 2022

Items	Before the intervention				After the intervention				
	Corrects		Errors		Corrects		Errors		*p* value
	n	%	n	%	n	%	n	%	
1.Do you know what standard precautions are?	90	90.9	9	9.1	97	98.0	2	2.0	0.035
2. Standard precautions should only be applied to patients with a diagnosed infection or patients who are in the incubation period for a particular infection.	64	64.6	35	35.4	72	72.7	27	27.3	0.182
3. Adherence to standard precautions aims primarily to protect healthcare staff.	15	15.2	83	83.8	29	29.3	70	70.7	0.013
4. When coming into contact with blood or any other potentially contaminated materials. hands should be washed immediately.	99	100.0	0	0.0	99	100.0	0	0.0	-
5. Hand hygiene should be performed during care provision to different patients.	91	91.9	8	8.1	92	92.9	7	7.1	0.763
6. Since the use of gloves can prevent hand contamination. it is not necessary to wash hands after removing gloves.	83	83.8	16	16.2	93	93.9	5	5.1	0.033
7. Contact with objects, materials, equipment, clothing, and individuals contaminated with PPE should be avoided.	89	89.9	7	7.1	91	91.9	8	8.1	0.637
8. PPE should not be shared.	94	94.9	5	5.1	92	92.9	7	7.1	0.564
9. When performing oral care procedures or other procedures that may involve contact with patient mucous membranes, the use of gloves is not mandatory.	85	85.9	13	13.1	92	92.9	6	6.1	0.09
10. Gloves are necessary in blood collection or venous puncture procedures.	99	100.0	0	0.0	97	98.0	1	1.0	0.157
11. Gloves are necessary in procedures where there is a possibility of hand contact with patient secretions or excretions.	98	99.0	1	1.0	99	100.0	0	0.0	0.317
12. Gloves should be changed when providing care to different patients.	98	99.0	0	0.0	97	98.0	0	0.0	0.564
13. A protective mask or face shield should be worn in procedures where there is a possibility of blood splashes, bodily fluids. secretions. or excretions.	94	94.9	4	4.0	97	98.0	1	1.0	0.257
14. Eye protection or face shields should be worn in procedures where there is a possibility of blood splashes, secretions, or excretions.	96	97.0	2	2.0	97	98.0	2	2.0	0.655
15. A protective gown should be worn in procedures where there is a possibility of blood splashes, bodily fluids, secretions, or excretions.	97	98.0	1	1.0	98	99.0	1	1.0	0.564
16. Disposable caps and shoe covers should be used in situations where there is a possibility of blood splashes, bodily fluids, secretions, or excretions	76	76.8	23	23.2	84	84.8	15	15.2	0.088
17. It is prohibited to bend, twist or actively cap needles. When necessary, passive capping should be done with only one hand. Disposal containers should be located close to the manipulation area.	83	83.8	15	15.2	86	86.9	13	13.1	0.549
18. When providing nursing care to patients with hepatitis C or syphilis only standard precautions need to be adopted.	59	59.6	40	40.4	65	65.7	34	34.3	0.330
19. When providing nursing care to patients with active tuberculosis or chickenpox, standard precautions, as well as droplet precautions, need to be adopted.	8	8.1	89	89.9	5	5.1	93	93.9	0.366
20. When providing nursing care to patients with intestinal infections or skin infections. contact precautions need to be adopted.	86	86.9	12	12.1	91	91.9	8	8.1	0.166

*Note: The unanswered items by each participant were considered as errors. Cochran's Q test.*

**Figure 2:**
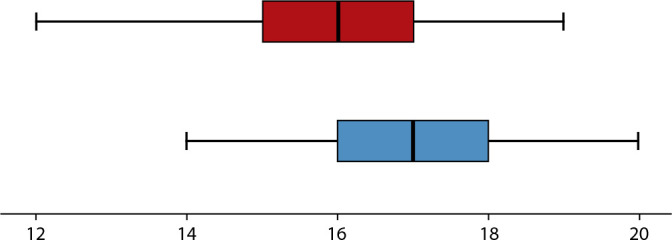
Box plot representing the participants’ grades before and after the intervention, Três Lagoas, Mato Grosso do Sul, Brazil, 2022

*Notes: Red: Before the intervention; Blue: After the intervention*

Below is Figure 2 represented by a box plot, where it is possible to observe a significant difference between the grades of healthcare professionals before (16.20 ± 1.51) and after (16.90 ± 1.31) the educational intervention (W = 3.336; p < 0.05). An increase in the score and a decrease in the range were observed for most questions before and after the educational intervention.

## DISCUSSION

We observed an increase in the average score of knowledge after an educational intervention aimed at improving the knowledge of nursing professionals about standard precautions. We also observed a decrease in the standard deviation, which suggests less dispersed scores among healthcare professionals before (16.20 ± 1.51) and after (16.90 ± 1.31) the intervention. However, this result contrasts with several studies^(25-27)^ in the literature showing knowledge gaps among professionals regarding standard precautions, reinforcing the need for constant training for the entire team^(28)^. Our findings are supported by another study conducted in São Paulo, Brazil, which diagnosed an improvement in the positive data of professionals’ knowledge after intervention^(29)^.

Although the context of standard precautions is broad, there is a significant improvement in the knowledge indicators concerning the purpose of standard precautions, moments for hand hygiene, indications for the use of gloves, shoe covers, and caps, and the PPE involved in the collection of biological material. After the educational intervention, the number of correct answers regarding the purpose of standard precautions doubled, and this increase was statistically significant. Although it may indicate a favorable scenario for knowledge change, this data is still concerning as it may minimize the importance of these measures due to improper or suboptimal use. The use of standard precautions, in addition to protecting professionals against occupational risks, also protects patients against possible infectious agents transported on the hands of professionals or equipment used^(25-29)^.

Regarding hand washing after using gloves, there was also a statistically significant increase in professionals’ knowledge. This result is particularly interesting considering the need to always improve good hand hygiene practices, as incorrect practices can favor the chain of cross-infection and compromise patients, families, and professionals, especially in the context of a health crisis like the current COVID-19^(26)^.

Since glove use prevents contamination, hand washing is indispensable regardless of suspicion or confirmation of infection between the care of different patients, and the understanding that glove use does not replace hand hygiene after care. According to the WHO, which has incorporated the “five moments for hand hygiene” approach for workers, hand hygiene is performed before and after touching a patient, before performing aseptic procedures, after exposure to body fluids, and after touching the patient’s environment, being one of the most important measures for infection prevention^(30)^.

The COVID-19 pandemic has made the need for correct hand washing even more critical in the prevention of viral infections. Therefore, for future pandemics, the need to strengthen these measures, including sanitary measures, will be of utmost importance in their containment and spread^(31)^.

Furthermore, regarding other types of precautions and when to use each PPE, we noted positive results regarding the protection of professionals in contact with patients with hepatitis C and syphilis. Before the intervention, 59.6% of professionals agreed that only standard precautions need to be adopted in these situations, and after the intervention, 65.7% of professionals demonstrated knowledge on this issue. This finding is supported by another intervention study conducted in Iran, which also showed a positive effect on professionals’ perception of the risk and severity of infection susceptibility to possible irreversible injuries resulting from contagion with these pathogens^(32)^.

On the other hand, regarding the adoption of droplet precautions and not aerosol precautions for assisting patients with active tuberculosis or chickenpox, 92 professionals continued to agree with the statement and 9.1% claimed they did not know which type of precaution to use when in contact with intestinal and skin infections, after an educational intervention. After the intervention, 5.1% of these professionals still had a lack of knowledge on the subject. We know the importance and necessity of knowing which personal protective equipment (PPE) to use and in which situations it is recommended, as this contributes to safe care for both the professional and the patient, reducing the occurrence of adverse events^(26-30)^.

However, after the educational intervention, we noticed a decrease in the percentage of professionals who considered it necessary to use gloves for blood collection, venous punctures, and whenever there is a possibility of hand contact with patient secretions, as well as an increase in patient exposure. This is concerning because the improper and indiscriminate use of gloves can be associated with the transmission of pathogenic agents^(33)^. On the other hand, there are reports in the literature about performing venous punctures without gloves^(34)^. Factors such as haste and talc were mentioned as reasons for not adhering to this PPE. Nevertheless, it is important to emphasize that individualized care for different patients is necessary to minimize the risk of infection and dissemination of microorganisms.

We observed that even with an increase in correct answers regarding the statement that needles should not be bent, twisted or actively capped, this practice still occurs among nursing professionals. Disposal containers should be placed close to the manipulation area, and there were professionals who continued to consider this statement false, suggesting that the practice of needle recapping is still present in their work activities^(35)^.

Precautions should be applied in the care of all patients^(19)^, regardless of diagnosis, and the critical scenario experienced during the COVID-19 pandemic seems to have amplified erroneous practices related to precautions, especially due to the lack of adequate knowledge. Although we recognize that knowledge alone is not sufficient to sustain a behavioral change, it remains a fundamental element for sustained behavioral change. Thus, nursing professionals can only carry out their activities with greater safety after acquiring the necessary knowledge^(36)^.

Some misconceptions demonstrate the lack of necessary knowledge and, consequently, increase the likelihood of occupational risks, leading to a higher risk of certain diseases for professionals. Therefore, knowledge about precautions includes safe and essential practices in the control of healthcare-associated infections, such as good hand hygiene, performed frequently, using not only water but also soap, as well as proper disposal of sharps and cleaning of surfaces and floors in case of blood spills^(37)^.

The importance of training involving biosafety practices is emphasized not only in the workplace, but also as a strategy to minimize the impacts resulting from a lack of knowledge about precautions since the professional’s preparation^(10)^. It is important to highlight that hospital control and organization need to be constant, especially after the pandemic, where considerable behavioral and care technique changes were observed, making it necessary to monitor the changes caused by COVID-19 closely, requiring greater coordination by management in ensuring the necessary resources and qualified workforce^(38)^.

### Limitations of the Study

The main limitations of this study are that it was conducted in a single hospital, which limits its generalizability. The fact that it relied solely on self-reporting and not on direct observation should also be considered. Lastly, it is worth noting that the focus on the knowledge aspect does not necessarily reflect the daily practical adherence to standard precautions by the healthcare professional during patient care.

### Contributions to the Nursing Field

The study demonstrates advancements for nursing science regarding the strengthening of existing knowledge and the understanding of new knowledge related to safe practices in healthcare settings. It allows for contributions to evidence-based practice, enabling a situational diagnosis of nursing professionals’ knowledge regarding the use of standard precautions after a pandemic outbreak, identifying knowledge deficits, and providing support for the development of targeted educational interventions. These interventions can serve as a foundation for the development of strategies after a public health crisis, such as the COVID-19 pandemic.

## CONCLUSION

The educational intervention proved to be effective, as an increase in the score of the Standard Precautions Knowledge Questionnaire and a decrease in the range for most questions were observed before and after the educational intervention. However, significant misunderstandings were still reported after the educational intervention, which suggests uncertainties among professionals in some of the topics covered. This could be related to the individual’s cognitive absorption capacity in understanding changes in a short period since the educational action was only appreciated by each participant once before the post-questionnaire reapplication for knowledge impact evaluation. This reflects the need for periodic and multimodal educational interventions. Therefore, the development of educational interventions with a defined methodological approach and the use of validated educational materials and tools led to knowledge improvement among the healthcare professionals.
